# Dataset concerning the mental health of healthcare professionals during COVID-19 pandemic in Bangladesh

**DOI:** 10.1016/j.dib.2021.107506

**Published:** 2021-10-23

**Authors:** Md. Rabiul Islam, Sumaiya Quaiyum, Sajuti Akter Pakhe, Md Azim Uddin Repon, Mohiuddin Ahmed Bhuiyan

**Affiliations:** Department of Pharmacy, University of Asia Pacific, 74/A Green Road, Farmgate, Dhaka 1215, Bangladesh

**Keywords:** Healthcare professionals, HCPs, Mental health, Loneliness, Depression, Anxiety, Sleep disturbance, COVID-19, Bangladesh

## Abstract

The present dataset is concerned with an article entitled “Effect of COVID-19 pandemic on mental health among Bangladeshi healthcare professionals: A cross-sectional study” [1]. This data article consists of a dataset regarding the mental health of Bangladeshi healthcare professionals (HCPs) during the ongoing COVID-19 pandemic. We collected the data from July 15, 2020, to September 20, 2020, using Google survey tools (Google Forms). A total of 355 complete responses have been obtained from the Bangladeshi HCPs aged between 20–60 years (male: 204, female: 151). We obtained informed consent from all participants for this study. We assessed the sociodemographic profile and four psychometric measures of HCPs working in Bangladesh during the COVID-19 pandemic. A structured questionnaire was used to collect the demographic information. We used the UCLA loneliness scale-8 (UCLA-8), the patient health questionnaire-9 (PHQ-9), the 7-item generalized anxiety disorder scale (GAD-7), and the Pittsburgh sleep quality index (PSQI) were applied to measure loneliness, depression, generalized anxiety, and sleep disturbance, respectively. This dataset presents the descriptive analysis of psychometric variables of participants. Also, the dataset might be used as a platform for future research on psychometric evaluation using the above scales. We included participants regardless of the sociodemographic backgrounds of HCPs. Therefore, the policymakers, government, and non-government organizations can use this data to develop different programs for promoting the mental health of HCPs and the general population in Bangladesh.

## Specifications Table

**Table:** 

Subject	Public Health.
Specific subject area	Mental health, psychology, and COVID-19.
Type of data	Primary data, tables, and figure.
How data were acquired	Google survey tools (Google Forms).
Data format	Raw and analyzed.
Parameters for data collection	We obtained responses from Bangladeshi healthcare professionals aged between 20 to 60 years. The target group was social media users who agreed to participate in this study regardless of their background or sociodemographic factors.
Description of data collection	We used Google survey tools (Google Forms) to conduct this study from July 15, 2020, to September 20, 2020. We collected responses from healthcare professionals (HCPs) using a self-administered structured questionnaire that included informed consent, sociodemographic information, and four distinct mental health assessment scales (UCLA-8, PHQ-9, GAD-7, and PSQI). We sent the questionnaires to the respondents through social media platforms and personal emailing. Also, we arranged phone calls to settle any concerns raised by the respondents. Moreover, we supplied the detailed questionnaire and responses as supplementary files 1 and 2.
Data source location	Researchers from the University of Asia Pacific, Dhaka, have collected data from across Bangladesh.
Data accessibility	Data is within this article.
Related research article	Repon, M., Pakhe, S. A., Quaiyum, S., Das, R., Daria, S., & Islam, M. R. (2021). Effect of COVID-19 pandemic on mental health among Bangladeshi healthcare professionals: A cross-sectional study. *Science progress*, *104*(2), 368504211026409. 10.1177/00368504211026409 https://journals.sagepub.com/doi/10.1177/00368504211026409

## Value of the Data

• Researchers can use this dataset to compare the mental health of other populations during this ongoing health emergency.
• The dataset allowed for quick data collection during global health emergencies.
• The present dataset can initiate studies to measure the severity of mental health problems among HCPs in Bangladesh to understand the gravity of the issue.
• The dataset is useful for policymakers and government or non-government organizations to take proper measures for promoting mental health.

## 1 Data Description

The World Health Organization (WHO) endorsed the COVID-19 as a global pandemic on March 11, 2020 [1], [2]. Since then, the world fighting this pandemic, and nobody knows when it will stop. The Bangladesh government declared a country-wide lockdown on March 26, 2020, to curb the spreading of this virus among its citizens [3]. The COVID-19 responses have impacted the personal, social, and work-life of many people across the globe [4], [5], [6], [7]. Also, the pandemic has more or less impact in every sector in a country [8], [9], [10]. Therefore, the COVID-19 pandemic tremendously affected the mental health of the general population of any country [11,12]. As frontline fighters, the impact of the COVID-19 pandemic on the mental health of HCPs was high due to this relatively unknown and lethal virus [13]. The ongoing pandemic has placed HCPs in a situation of intense psychological pressure and moral responsibilities [14].

According to the findings, the composition of physicians, pharmacists, nurses, and medical technologists were 30%, 23%, 26%, and 22%, respectively. Among all HCPs, males and females were 57 and 43%. Only 36% HCPs were above 40 years of age, and the majority of the respondents were from a lower economic class (72%) residing in the urban area (76%).

**Fig. 1:**
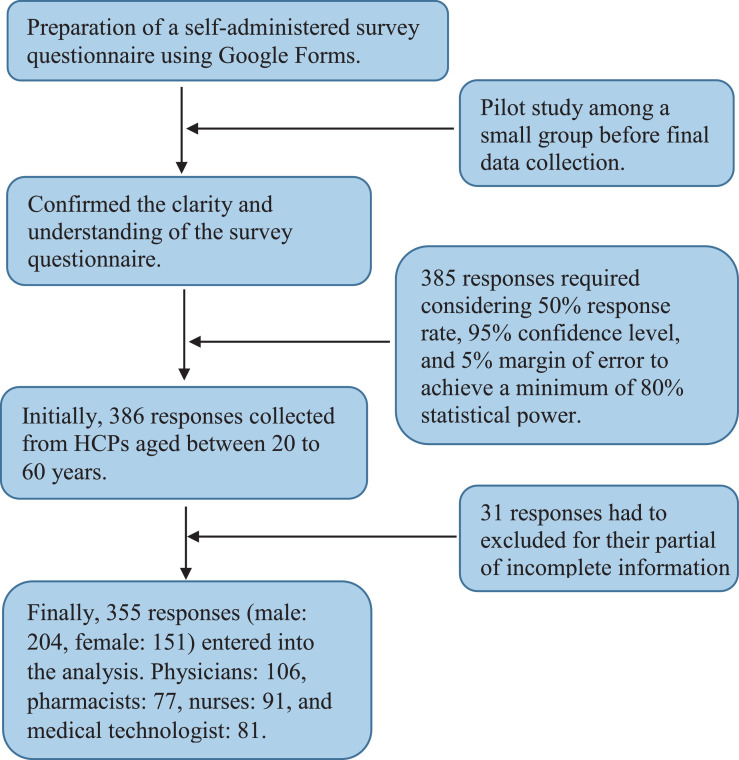
Flowchart of collecting responses from the participants.

The dataset provides (i) evaluation of loneliness by using the UCLA loneliness scale-8 (UCLA-8) in Table 1, (ii) evaluation of depression by the patient health questionnaire-9 (PHQ-9) in Table 2, (iii) evaluation of anxiety applying the 7-item generalized anxiety disorder scale (GAD-7) in Table 3, (iv) evaluation of sleep disturbances by the Pittsburgh sleep quality index (PSQI) in Table 4, (v) distribution of different psychometric parameters among the respondents in Table 5, (vi) the severity of different psychometric parameters among the respondents in Table 6. We presented a detailed data collection procedure using a flowchart (Fig. 1). Also, we provided the questionnaire and raw data as supplementary files (supplementary file-1 and file-2, respectively).

**Table 1:** Distribution of responses based on the UCLA loneliness scale-8 (UCLA-8).

Indicate how often each of the statements below is		
descriptive of you.	Frequency (n)	Percentage (%)
1. In the past 30 days, I lack companionship.		
Never (1)	42	11.83
Rarely (2)	47	13.24
Sometimes (3)	167	47.04
Often (4)	99	27.89
2. In the past 30 days, there is no one I can turn to.		
Never (1)	70	19.72
Rarely (2)	63	17.75
Sometimes (3)	155	43.66
Often (4)	67	18.87
3. In the past 30 days, I feel left out.		
Never (1)	68	19.15
Rarely (2)	69	19.44
Sometimes (3)	154	43.38
Often (4)	64	18.03
4. In the last 30 days, I feel isolated from others.		
Never (1)	55	15.49
Rarely (2)	65	18.32
Sometimes (3)	144	40.56
Often (4)	91	25.63
5. In the last 30 days, I am unhappy being so withdrawn.		
Never (1)	53	14.93
Rarely (2)	68	19.16
Sometimes (3)	172	48.45
Often (4)	62	17.46
6. In the last 30 days, people are around me but not with me.		
Never (1)	44	12.39
Rarely (2)	104	29.30
Sometimes (3)	115	32.39
Often (4)	92	25.92
7. In the last 30 days, I am an outgoing person.		
Never (1)	36	10.14
Rarely (2)	104	29.30
Sometimes (3)	137	38.59
Often (4)	78	21.97
8. In the last 30 days, I can find companionship when I want it.		
Never (1)	21	5.92
Rarely (2)	120	33.80
Sometimes (3)	143	40.28
Often (4)	71	20.00

**Table 2:** Distribution of responses based on the patient health questionnaire-9 (PHQ-9).

Indicate how often each of the statements below is descriptive of you.	Frequency (n)	Percentage (%)
1. In the last two weeks, little interest or pleasure in doing things.		
Not at all (0)	105	29.58
Several days (1)	143	40.28
More than half of the days (2)	52	14.65
Nearly every day (3)	55	15.49
2. In the last two weeks, feeling down, depressed or hopeless.		
Not at all (0)	105	29.58
Several days (1)	143	40.28
More than half of the days (2)	52	14.65
Nearly every day (3)	55	15.49
3. In the last two weeks, trouble falling or staying asleep, sleeping too much.		
Not at all (0)	102	28.73
Several days (1)	164	46.20
More than half of the days (2)	50	14.08
Nearly every day (3)	39	10.99
4. In the last two weeks, feeling tired or having little energy.		
Not at all (0)	83	23.38
Several days (1)	166	46.76
More than half of the days (2)	60	16.90
Nearly every day (3)	46	12.96
5. In the last two weeks, poor appetite or overeating.		
Not at all (0)	103	29.01
Several days (1)	167	47.04
More than half of the days (2)	57	16.06
Nearly every day (3)	28	7.89
6. In the last two weeks, feeling bad about yourself-or that you are a failure or have let yourself or your family down.		
Not at all (0)	116	32.68
Several days (1)	151	42.54
More than half of the days (2)	50	14.08
Nearly every day (3)	38	10.70
7. In the last two weeks, trouble concentrating on things, such as reading the newspaper or watching television.		
Not at all (0)	102	28.73
Several days (1)	137	38.59
More than half of the days (2)	82	23.10
Nearly every day (3)	34	9.58
8. In the last two weeks, moving or speaking so slowly or the opposite-moving around a lot more than usual.		
Not at all (0)	146	41.13
Several days (1)	130	36.62
More than half of the days (2)	49	13.80
Nearly every day (3)	30	8.45
9. In the last two weeks, thoughts that you would be better off dead, or of hurting yourself in some way.		
Not at all (0)	175	49.30
Several days (1)	124	34.93
More than half of the days (2)	27	7.61
Nearly every day (3)	29	8.16

**Table 3:** Distribution of responses based on the 7-item generalized anxiety disorder (GAD-7) scale.

Indicate how often each of the statements below is descriptive of you.	Frequency (n)	Percentage (%)
1. In the last two weeks, I am feeling nervous, anxious, or on edge.		
Not at all (0)	79	22.25
Several days (1)	146	41.13
More than half of the days (2)	60	16.90
Nearly every day (3)	70	19.72
2. In the last two weeks, I am not being able to stop or control worrying.		
Not at all (0)	79	22.25
Several days (1)	147	41.42
More than half of the days (2)	62	17.46
Nearly every day (3)	67	18.87
3. In the last two weeks, I am worrying too much about different things.		
Not at all (0)	70	19.72
Several days (1)	156	43.94
More than half of the days (2)	58	16.34
Nearly every day (3)	71	20.00
4. In the last two weeks, I felt trouble in relaxing.		
Not at all (0)	102	28.73
Several days (1)	150	42.25
More than half of the days (2)	46	12.96
Nearly every day (3)	57	16.06
5. In the last two weeks, I am being so restless that it's hard to sit still.		
Not at all (0)	108	30.42
Several days (1)	132	37.19
More than half of the days (2)	67	18.87
Nearly every day (3)	48	13.52
6. In the last two weeks, I becoming easily annoyed or irritable.		
Not at all (0)	73	20.56
Several days (1)	151	42.54
More than half of the days (2)	72	20.28
Nearly every day (3)	59	16.62
7. In the last two weeks, I am feeling afraid as if something awful might happen.		
Not at all (0)	78	21.97
Several days (1)	143	40.28
More than half of the days (2)	39	10.99
Nearly every day (3)	95	26.76

**Table 4:** Distribution of responses based on the Pittsburgh sleep quality index (PSQI).

Indicate how often each of the statements below is descriptive of you.	Frequency (n)	Percentage (%)
1. During the past month, when have you usually gone to bed at night?		
Before 10.00 PM	4	1.13
10.01 PM to 12.00 AM	116	32.68
12.01 AM to 2.00 AM	199	56.05
After 2.00 AM	36	10.14
2. During the past month, how long (in minutes) has it usually take you to fall asleep each night?		
Less than 15 minutes	0	0.00
15-30 minutes	315	88.73
31-60 minutes	22	6.20
More than 60 minutes	18	5.07
3. During the past month, when have you usually gotten up in the morning?		
Before 5.00 AM	48	13.52
5.00 AM to 7.00 AM	155	43.66
7.01 AM to 9.00 AM	119	33.52
After 9.00 AM	33	9.30
4. During the past month, how many hours of actual sleep did you get at night?		
Less than 4 hours	6	1.69
4 to 6 hours	290	81.69
7 to 8 hours	58	16.34
More than 8 hours	1	0.28
5. During the past month, how many hours do you spend in bed?		
Less than 5 hours	55	15.49
5 to 7 hours	245	69.02
8 to 10 hours	53	14.93
More than 10 hours	2	0.56
6. During the past month, how many times, you cannot get to sleep within 30 minutes?		
Not during last month (0)	88	24.79
Less than once a week (1)	133	37.47
Once or twice a week (2)	96	27.04
Three or more in week (3)	38	10.70
7. During the past month, how many times, you wake up in the middle of the night or early morning?		
Not during last month (0)	73	20.57
Less than once a week (1)	143	40.28
Once or twice a week (2)	107	30.14
Three or more in week (3)	32	9.01
8. During the past month, how many times, you have to get up to use the bathroom?		
Not during last month (0)	104	29.30
Less than once a week (1)	121	34.08
Once or twice a week (2)	87	24.51
Three or more in week (3)	43	12.11
9. During the past month, how many times, you cannot breathe comfortably?		
Not during last month (0)	154	43.38
Less than once a week (1)	106	29.86
Once or twice a week (2)	77	21.69
Three or more in week (3)	18	5.07
10. During the past month, how many times, you cough or snore loudly?		
Not during last month (0)	122	34.37
Less than once a week (1)	132	37.18
Once or twice a week (2)	81	22.82
Three or more in week (3)	20	5.63
11. During the past month, how many times, you feel too cold?		
Not during last month (0)	155	43.66
Less than once a week (1)	115	32.39
Once or twice a week (2)	75	21.13
Three or more in week (3)	10	2.82
12. During the past month, how many times, you feel too hot?		
Not during last month (0)	111	31.27
Less than once a week (1)	87	24.50
Once or twice a week (2)	111	31.27
Three or more in week (3)	46	12.96
13. During the past month, how many times, you had bad dreams?		
Not during last month (0)	95	26.76
Less than once a week (1)	140	39.44
Once or twice a week (2)	96	27.04
Three or more in week (3)	24	6.76
14. During the past month, how many times, you have pain during sleep?		
Not during last month (0)	132	37.18
Less than once a week (1)	105	29.58
Once or twice a week (2)	91	25.63
Three or more in week (3)	27	7.61
15. During the past month, how many times, you have trouble in sleeping because of any other reasons?		
Not during last month (0)	108	30.42
Less than once a week (1)	124	34.93
Once or twice a week (2)	106	29.86
Three or more in week (3)	17	4.79
16. During the past month, how often have you taken medicine to help you sleep?		
Not during last month (0)	189	53.24
Less than once a week (1)	88	24.79
Once or twice a week (2)	64	18.03
Three or more in week (3)	14	3.94
17. During the past month, how many times you did not sleep due to any program or other important case?		
Not during last month (0)	107	30.14
Less than once a week (1)	124	34.93
Once or twice a week (2)	106	29.86
Three or more in week (3)	18	5.07
18 .During the past month, how much of a problem has it been for you to keep up enough enthusiasm to get things done?		
Not during last month (0)	113	31.83
Less than once a week (1)	140	39.44
Once or twice a week (2)	76	21.41
Three or more in week (3)	26	7.32
19. During the past month, how would you rate your sleep quality overall?		
Very good (0)	53	14.93
Fairly good (1)	194	54.65
Fairly bad (2)	90	25.35
Very bad (3)	18	5.07

**Table 5:** Different psychometric parameters among the respondents.

Psychometric parameters (total responses, *N* = 355)	Frequency (n)	Percentage (%)
Loneliness		
Yes	315	88.73
No	40	11.27
Depression		
Yes	158	44.51
No	197	55.49
Generalized anxiety		
Yes	276	77.75
No	79	22.25
Sleep disturbance		
Yes	308	86.76
No	47	13.24

**Table 6:** Severity of different psychometric parameters among the respondents.

Psychometric parameters (total responses, N=355)	Frequency (n)	Percentage (%)
*Loneliness*	*315*	*88.73*
Mild	115	36.50
Moderate	160	50.79
Severe	40	12.71
*Depression*	*158*	*44.50*
Mild	103	65.19
Moderate	45	28.48
Severe	10	6.33
*Generalized anxiety*	*276*	*77.75*
Mild	118	42.75
Moderate	106	38.41
Severe	52	18.84
*Sleep disturbance*	*308*	*86.76*
Mild	189	61.36
Moderate	104	33.77
Severe	15	4.87

## 2 Experimental Design, Materials, and Methods

We conducted a nationwide online survey to investigate the effects of the COVID-19 pandemic on mental health among Bangladeshi HCPs. We collected responses between July 15, 2020, and September 20, 2020, using Google Forms. Respondents got the questionnaire link via different social media and personal emailing. However, we conducted a pilot study for clarity and understanding of the procedure. Also, we communicated with the respondents through phone calls or video conferencing to solve any confusion regarding the questionnaire during data collection. We designed the Google Forms in a way that we can avoid multiple responses from a single respondent. Also, we tracked the IP addresses of the individual respondents to ensure data sanity. We divided the questionnaire into two major sections: (1) questionnaire about sociodemographic, (2) questionnaire about psychometric measures. We used a structured questionnaire to collect demographic information, and we applied four internationally validated psychometric scales to assess mental health. We used UCLA-8, PHQ-9, GAD-7, and PSQI to assess loneliness, depression, generalized anxiety disorder, and sleep problems, respectively [15], [16], [17], [18]. We used every scale entirely, and we did not change anything on those scales. We measured loneliness by eight questions on how frequently they felt the following statements in the last 30 days. Respondents rated each question from 1 to 4 based on the answer: 1 (never), 2 (rarely), 3 (sometimes), and 4 (always) (often). Then there were nine questions concerning how frequently the respondents had been concerned by any of the following difficulties in the previous 30 days to evaluate depression level. Participants rated all the questions related to depression from 0 to 3 based on the response: 0 (not at all), 1 (several days), 2 (more than half of the days), and 3 (nearly every day). Psychometric measures included seven questions indicating how frequently the respondents were affected by the following issues in the last 30 days to assess respondents' anxiety levels. The responses range from 0 to 3 scale based on the answer: 0 (not at all), 1 (several days), 2 (more than half of the days), and 3 (nearly every day). Finally, we assessed the sleep disturbance by asking nineteen questions. The responses of these nineteen questions were then grouped into seven areas to measure the overall sleep quality. We performed data analyzes using Microsoft excel 2016 and presented them as frequency and percentage.

## Funding

The author(s) did not receive any financial support for this work.

## Ethics Statement

We obtained approval of this study protocol from the ethical review committee of the department of psychiatry, Bangabandhu Sheikh Mujib Medical University, Dhaka, Bangladesh (2020/3609). Also, we obtained informed consent from all participants for their participation in this study and publication of anonymous data in the journal article.

## CRediT authorship contribution statement

**Md. Rabiul Islam:** Data curation, Conceptualization, Formal analysis, Writing – original draft, Supervision, Writing – review & editing. **Sumaiya Quaiyum:** Data curation, Conceptualization, Formal analysis, Writing – original draft. **Sajuti Akter Pakhe:** Data curation, Conceptualization, Formal analysis, Writing – original draft. **Md Azim Uddin Repon:** Data curation, Conceptualization, Formal analysis, Writing – original draft. **Mohiuddin Ahmed Bhuiyan:** Supervision, Writing – review & editing, Data curation.

## Declaration of Competing Interest

The author(s) do not have any conflict of interest to declare.

## Appendix Supplementary materials

Image, application 1

The survey questionnaire and the responses to the questions in English have been supplied as supplementary file 1 and 2. Image, application 2
