# Supplementary material for: Dataset concerning the mental health of healthcare professionals during COVID-19 pandemic in Bangladesh
Source: Data Brief. 2021 Oct 23;39:107506. doi: 10.1016/j.dib.2021.107506 (PMC8553544; doi:10.1016/j.dib.2021.107506)
Supplement: Supplementary file 1 [file mmc1.docx]

A survey on mental health status among Bangladeshi healthcare professionals during the COVID-19 pandemic

**Please tick one box for each statement**

*Section 1: Demographic questions*

*Express your consent to participate in the research and processing of anonymous data for scientific purposes.

- - Agree

1. Profession

- Physician
- Pharmacist
- Nurse
- Medical technologist

1. *Age range in years

- 20-30
- 31-40
- 41-50
- 51-60

1. *Gender

- Male
- Female

1. *Weight and height ratio/BMI score
   - Below 18.5
   - 18.5–25.0
   - Above 25.0
2. *Marital status
   - Married
   - Unmarried
3. *Education
   - Higher secondary level
   - Graduate/above
4. * Work place
   - Hospital/clinic
   - Private chamber
   - Pharma industry
   - Others
5. *Economic status
   - Low
   - Medium
   - High
6. *Residence
   - Urban area
   - Rural area
7. *Living status
   - With family
   - Without family
8. *Smoking habit
   - Smoker
   - Non-smoker
9. *Religion
   - Muslim
   - Hindu
   - Others

*Section 2: Psychometric assessment*

Indicate how often each of the statements below is descriptive of you.

**Loneliness Scale (UCLA-8)**

1. *In the past 30 days, I lack companionship.
   - Never (0)
   - Rarely (1)
   - Sometimes (2)
   - Often (3)
2. *In the past 30 days, there is no one I can turn to.
   - Never (0)
   - Rarely (1)
   - Sometimes (2)
   - Often (3)
3. *In the past 30 days, I feel left out.
   - Never (0)
   - Rarely (1)
   - Sometimes (2)
   - Often (3)
4. *In the last 30 days, I feel isolated from others.
   - Never (0)
   - Rarely (1)
   - Sometimes (2)
   - Often (3)
5. *In the last 30 days, I am unhappy being so withdrawn.
   - Never (0)
   - Rarely (1)
   - Sometimes (2)
   - Often (3)
6. *In the last 30 days, people are around me but not with me.
   - Never (0)
   - Rarely (1)
   - Sometimes (2)
   - Often (3)
7. *In the last 30 days, I am an outgoing person.
   - Never (0)
   - Rarely (1)
   - Sometimes (2)
   - Often (3)
8. *In the last 30 days, I can find companionship when I want it.
   - Never (0)
   - Rarely (1)
   - Sometimes (2)
   - Often (3)

**Patient Health Questionnaire-9 (PHQ-9)**

1. *In the last two weeks, little interest or pleasure in doing things.
   - Not at all (0)
   - Several days (1)
   - Half of days (2)
   - Nearly every day (3)
2. *In the last two weeks, feeling down, depressed or hopeless.
   - Not at all (0)
   - Several days (1)
   - Half of days (2)
   - Nearly every day (3)
3. *In the last two weeks, trouble falling or staying asleep, sleeping too much
   - Not at all (0)
   - Several days (1)
   - Half of days (2)
   - Nearly every day (3)
4. *In the last two weeks, feeling tired or having little energy.
   - Not at all (0)
   - Several days (1)
   - Half of days (2)
   - Nearly every day (3)
5. *In the last two weeks, poor appetite or over-eating.
   - Not at all (0)
   - Several days (1)
   - Half of days (2)
   - Nearly every day (3)
6. *In the last two weeks, feeling bad about yourself-or that you are a failure or have let yourself or your family down.
   - Not at all (0)
   - Several days (1)
   - Half of days (2)
   - Nearly every day (3)
7. *In the last two weeks, trouble concentrating on things, such as reading the newspaper or watching television.
   - Not at all (0)
   - Several days (1)
   - Half of days (2)
   - Nearly every day (3)
8. *In the last two weeks, moving or speaking so slowly or the opposite-moving around a lot more than usual.
   - Not at all (0)
   - Several days (1)
   - Half of days (2)
   - Nearly every day (3)
9. *In the last two weeks, thoughts that you would be better off dead, or of hurting yourself.
   - Not at all (0)
   - Several days (1)
   - Half of days (2)
   - Nearly every day (3)

**Generalized Anxiety Disorder Scale (GAD-7)**

1. *In the last two weeks, I am feeling nervous, anxious, or on edge.
   - Not at all (0)
   - Several days (1)
   - More than half of the days (2)
   - Nearly every day (3)
2. *In the last two weeks, I am not being able to stop or control worrying.
   - Not at all (0)
   - Several days (1)
   - More than half of the days (2)
   - Nearly every day (3)
3. *In the last two weeks, I am worrying too much about different things.
   - Not at all (0)
   - Several days (1)
   - More than half of the days (2)
   - Nearly every day (3)
4. *In the last two weeks, I feel trouble in relaxing.
   - Not at all (0)
   - Several days (1)
   - More than half of the days (2)
   - Nearly every day (3)
5. *In the last two weeks, I am being so restless that it's hard to sit still.
   - Not at all (0)
   - Several days (1)
   - More than half of the days (2)
   - Nearly every day (3)
6. *In the last two weeks, I becoming easily annoyed or irritable.
   - Not at all (0)
   - Several days (1)
   - More than half of the days (2)
   - Nearly every day (3)
7. *In the last two weeks, I am feeling afraid as if something awful might happen.
   - Not at all (0)
   - Several days (1)
   - More than half of the days (2)
   - Nearly every day (3)

**Pittsburgh Sleep Quality Index**

1. * During the past month, when have you usually gone to bed at night?
   - Before 10.00 PM
   - 10.01 PM to 12.00 AM
   - 12.01 AM to 2.00 AM
   - After 2.00 AM
2. * During the past month, how long (in minutes) has it take you to fall asleep each night?

- Less than 15 minutes
- 15-30 minutes
- 31-60 minutes
- More than 60 minutes

1. * During the past month, when have you usually gotten up in the morning?

- Before 5.00 AM
- 5.00 AM to 7.00 AM
- 7.01 AM to 9.00 AM
- After 9.00 AM

1. * During the past month, how many hours of actual sleep did you get at night?

- Less than 4 hours
- 4 to 6 hours
- 7 to 8 hours
- More than 8 hours

1. * During the past month, how many hours do you spend in bed?

- Less than 5 hours
- 5 to 7 hours
- 8 to 10 hours
- More than 10 hours

1. * During the past month, how many times, you cannot get to sleep within 30 minutes?

- Not during last month (0)
- Less than once a week (1)
- Once or twice a week (2)
- Three or more in week (3)

1. * During the past month, how many times, you wake up in the middle of the night or early morning?

- Not during last month (0)
- Less than once a week (1)
- Once or twice a week (2)
- Three or more in week (3)

1. * During the past month, how many times, you have to get up to use the bathroom?

- Not during last month (0)
- Less than once a week (1)
- Once or twice a week (2)
- Three or more in week (3)

1. * During the past month, how many times, you cannot breathe comfortably?

- Not during last month (0)
- Less than once a week (1)
- Once or twice a week (2)
- Three or more in week (3)

1. * During the past month, how many times, you cough or snore loudly?

- Not during last month (0)
- Less than once a week (1)
- Once or twice a week (2)
- Three or more in week (3)

1. * During the past month, how many times, you feel too cold?

- Not during last month (0)
- Less than once a week (1)
- Once or twice a week (2)
- Three or more in week (3)

1. * During the past month, how many times, you feel too hot?

- Not during last month (0)
- Less than once a week (1)
- Once or twice a week (2)
- Three or more in week (3)

1. * During the past month, how many times, you had bad dreams?

- Not during last month (0)
- Less than once a week (1)
- Once or twice a week (2)
- Three or more in week (3)

1. * During the past month, how many times, you have pain during sleep?

- Not during last month (0)
- Less than once a week (1)
- Once or twice a week (2)
- Three or more in week (3)

1. * During the past month, how many times, you have trouble in sleeping because of any other reason?

- Not during last month (0)
- Less than once a week (1)
- Once or twice a week (2)
- Three or more in week (3)

1. * During the past month, how often have you taken medicine to help you sleep?

- Not during last month (0)
- Less than once a week (1)
- Once or twice a week (2)
- Three or more in week (3)

1. * During the past month, how many times you did not sleep due to any program or other important case?

- Not during last month (0)
- Less than once a week (1)
- Once or twice a week (2)
- Three or more in week (3)

18. * During the past month, how much of a problem has it been for you to keep up enough enthusiasm to get things done?

- Not during last month (0)
- Less than once a week (1)
- Once or twice a week (2)
- Three or more in week (3)

19. * During the past month, how would you rate your sleep quality overall?

- Very good (0)
- Fairly good (1)
- Fairly bad (2)
- Very bad (3)

Any comments

……………………………………………………………………………………………………..

Thank you for completing this survey

*Mandatory Questions

Tip: The questionnaire includes skip Logic questions
